# Candidate Molecular Compounds as Potential Indicators for Meibomian Gland Dysfunction

**DOI:** 10.3389/fmed.2022.873538

**Published:** 2022-05-24

**Authors:** Kofi Asiedu

**Affiliations:** ^1^Eye Clinic, Cosmopolitan Medical Centre, Accra, Ghana; ^2^School of Optometry and Vision Science, Faculty of Medicine and Health, The University of New South Wales, Sydney, NSW, Australia

**Keywords:** meibum, glands, lipids, interleukins, biomarkers

## Abstract

Meibomian gland dysfunction (MGD) is the leading cause of dry eye disease throughout the world. Studies have shown that several molecules in meibum, including but not limited to interleukins, amino acids, cadherins, eicosanoids, carbohydrates, and proteins, are altered in meibomian gland dysfunction compared with healthy normal controls. Some of these molecules such as antileukoproteinase, phospholipase A2, and lactoperoxidase also show differences in concentrations in tears between meibomian gland dysfunction and dry eye disease, further boosting hopes as candidate biomarkers. MGD is a complex condition, making it difficult to distinguish patients using single biomarkers. Therefore, multiple biomarkers forming a multiplex panel may be required. This review aims to describe molecules comprising lipids, proteins, and carbohydrates with the potential of serving various capacities as monitoring, predictive, diagnostic, and risk biomarkers for meibomian gland dysfunction.

## Introduction

The latest definition of meibomian gland dysfunction (MGD) as proposed by the MGD workshop 2011 is “Meibomian gland dysfunction is a chronic, diffuse abnormality of the meibomian glands, commonly characterized by terminal duct obstruction and/or qualitative/quantitative changes in the glandular secretion. It may result in alteration of the tear film, symptoms of eye irritation, clinically apparent inflammation, and ocular surface disease” (1).

The postulated pathomechanism underpinning the most common form of MGD is obstruction of the meibomian gland orifices as a result of hyperkeratinization of the ductal epithelium perpetuating the blockade of the gland orifices, stagnation of the gland contents, cystic dilatation, and then the miniaturization of the holocrine, secretory acini (2, 3). Researchers worldwide are making attempts to understand the pathomechanisms associated with the insurgence of MGD and the factors that make the condition chronic (4–7). Despite the increasing number of interventional studies on MGD (8–10), only a few new treatments have emerged (11), not one fix for all. Still, clinicians have to combine several treatments to address various aspects of this chronic condition (12). Biomarkers may facilitate the much-needed and anticipated personalized treatment for MGD, which is currently elusive. Current treatment options are often trial and error. Interventional studies on MGD have suffered from the lack of validated and specific biomarkers obscuring objectivity and endpoints’ reproducibility (12).

“A biomarker is defined as a characteristic that is measured objectively and evaluated as an indicator of normal biological processes, pathogenic processes, or biological responses to a therapeutic intervention” (13). Biomarkers aren’t universal and can be classified as diagnostic biomarkers, monitoring biomarkers, predictive biomarkers, safety biomarkers, risk biomarkers, pharmacodynamic biomarkers, and others (14, 15). As reiterated by the BEST Resource FDA-NIH Biomarker Working Group, biomarkers should be objective, reproducible, and a continuous measure (16). A biomarker is not an evaluator of how a subject feels, functions, or survives (13). A surrogate endpoint, on the contrary, is defined as “an endpoint that is used in clinical trials as a direct measure of how a patient feels, functions, or survives” (17). It is never intended to measure the clinical benefit of primary interest in and of itself; instead, it’s intended to foretell the clinical benefit or harm based on evidence. Besides these, a biomarker should have excellent specificity, sensitivity, reliability, and reproducibility, and, more importantly, be less expensive (18).

In recent times, imaging of the meibomian glands *via* meibography is commonplace and can be included in interventional studies (19, 20), but may not be sufficient for the diagnosis of MGD. Further work will be needed to validate mechanisms of analysis, including the development of automated systems and the correlation of meibomian gland changes with clinical findings (4). Several studies have endeavored to enrich the literature on various molecules in meibum and tear film of MGD subjects compared with controls. This review aims to describe molecules comprising lipids, proteins, and carbohydrates with the potential of serving various capacities as monitoring, predictive, diagnostic, and risk biomarkers for meibomian gland dysfunction.

## Methods

Peer-reviewed articles were searched from PubMed and Embase with no limitation on duration. For PubMed, the following search strategy was used, incorporating Text Words (TW) and Medical Subject Headings (MeSH), and combining appropriate terms using Boolean logic operators: Biomarker: “biomarker’s” [All Fields] OR “biomarkers” [MeSH Terms] OR “biomarkers” [All Fields] OR “biomarker” [All Fields] meibomian glands: “meibomian glands” [MeSH Terms] OR (“meibomian” [All Fields] AND “glands” [All Fields]) OR “meibomian glands” [All Fields]. The same approach was used in Embase-Ovid using the same search strategy. Each article’s title and abstract were read to determine their eligibility for inclusion. Other papers not appearing in the search were included by searching the reference list of some important papers. Publications were included in the current review if they were centered on molecules capable of serving as biomarkers for MGD. A total of 111 articles were related to the subject and were included in the review.

## Proteomic and Lipidomic Analysis of Meibum

The lipid layer is the foundational bedrock for tear film stability (21), which has made altered lipid layer thickness (LLT) a good indicator of MGD (4, 18, 22). The functional network analysis has created a platform for investigating the main biological processes needed to reveal candidate biomarkers (23). The studies of differential protein expression in complex biofluids such as tear film require rapid, highly reproducible, and accurate quantification (23, 24). Tear film proteomics and lipidomics offer robust analytical apparatus for assessing proteins and lipids involved in the pathogenesis of MGD. New emerging proteomics technologies such as the Tags for Relative and Absolute Quantitation (iTRAQ), the differential in-gel electrophoresis (DIGE), stable isotope labeling with amino acids in cell culture (SILAC), isotope-codes affinity tag (iCAT), absolute protein quantitation (AQUA), mass spectrometric technology, and label-free quantification (LFQ) have empowered research scientists to use tear proteomics to compare changes within normal tears allowing the elucidation of candidate proteins involved in the pathogenesis of MGD (25, 26).

**FIGURE 1:**
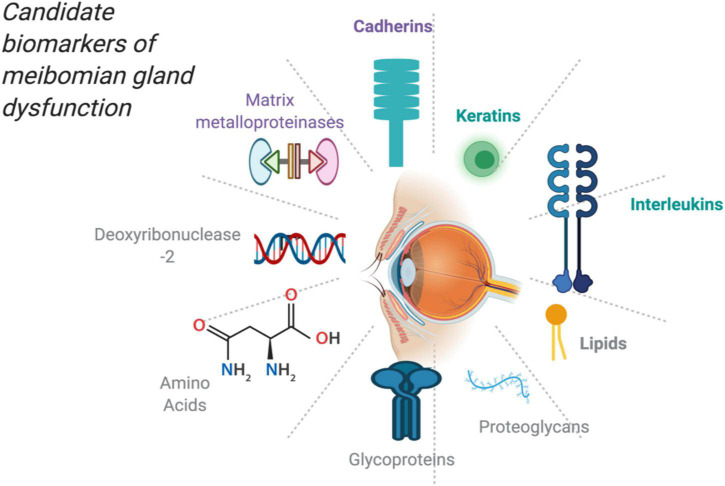
Potential and candidate molecules as biomarkers for MGD created with publication license from BioRender.com.

Using these analytical apparatus, it has become increasingly feasible to determine the changes in these proteins and lipids in the fundamental pathophysiological processes and issuing potential candidate biomarkers (23). This is illustrated in Figure 1 showing potential molecules that could serve as biomarkers upon further research.

## Lipids in Meibum

The amount of meibum on the lid margin is more than the amount required to form a 17-molecule thick lipid layer on the tears’ surface (24). Studies suggest that if there are patent meibomian gland orifices, it depend on the quality and not the lipid quantity for a stable tear film (24). Meibum comprises non-polar lipids (e.g., cholesterol esters, monoglycerides, triglycerides, wax esters, diesters, diglycerides, free fatty acids, and hydrocarbons) and polar lipids (e.g., ω-hydroxy fatty acids, sphingophospholipids, and glycerophospholipids) (18, 27–30). The lipid layer of the tear film has a classic duplex structure, comprising non-polar lipids (e.g., cholesterol esters, diesters, and wax esters) that form a lipophilic layer at the air-lipid interface and polar lipids (e.g., phospholipids) that differentiate the tear film lipid layer from the muco-aqueous layer interface (31, 32). It has been shown that there is significant variation in meibum composition among normal subjects (33). Butovich et al. showed high similarities of lipids in meibum between two different age groups, with only minor changes in the specific lipid species (34). The extent of the intergroup variability for tested lipid species was comparable to the intragroup variability for the same meibum components (34). No statistically significant differences in the lipid esterification, elongation, and unsaturation patterns were noted or documented. Aging itself seems to have only a minor effect on meibogenesis in healthy individuals without MGD or dry eye disease (34).

## Proteins in Meibum

More than 90 proteins have been identified in human meibum. They comprise keratins (K1, 5, 6, 7, 9, 10, 13, 16), surfactant proteins (SP-B, SP-C), lipocalins, lactoferrin, phospholipid transfer proteins, lipophillins, cytochrome *c*, farnesoid X laminin α-3 chain, lysozyme c, and proteoglycans, which are considered significant protein constituents of meibum (35).

## Lipids and Fatty Acids as Candidate Biomarkers for Meibomian Gland Dysfunction

Many investigators have found significant differences such as fatty acid composition between the meibum obtained from normal subjects and that obtained from subjects with blepharitis or dry eye disease (36–38). In meibum synthesis and subsequent release into the tear film, basal meibocytes move during their maturation, from the basal compartment of the acinus toward its center (39) and eventually toward the entrance of the ductule, which simultaneously occurs with the production and accumulation of lipids. MGD is a complex disease that involves several inflammatory pathways that contribute to the vicious cycle of the disease (40). Terminal duct obstruction, a core mechanism of MGD, usually creates an elevated intraglandular pressure and acinar epithelial stress that instigate the release of proinflammatory mediators such as cytokines and the subsequent clinically apparent inflammation seen in MGD (40).

One feature of obstructive MGD is that terminal ducts are obstructed, which leads to prolonged intraglandular accumulation of lipids with extensive enzymatic changes, leading to increased lipid mediators in the meibum (41, 42). The result of these enzymatic changes is an elevated concentration of lipid mediators in tears (41). In fact, researchers found that a tear film panel comprising 5-hydroxyeicosatetraenoic acid, leukotriene B4, and 18-hydroxyeicosapentaenoic acid was predictive of reduced meibum expressibility (41).

In MGD, the meibomian glands secrete abnormal lipids that cause excessive evaporation of the tear fluid. Linoleic acid is commonly associated with meibomian sterol and wax esters (43), and the elevated free linoleic acid concentration in the meibum of patients with MGD may be the reflection of increased content of linoleic acid chains within the non-polar lipids. The linoleic acid occurs as a free molecule or as an ester moiety, and its increment in meibum is responsible for the disruption of tight molecular packing within the tear film’s lipid layer, which makes the lipid layer lose the ability to mitigate aqueous tear evaporation (44).

Although linoleic acid occurs in the normal meibum, elevated linoleic acid is associated with MGD (42). Arita et al. reported that the relative amount of linoleic acid in meibomian gland secretion is associated with the severity of plugging of gland orifices and telangiectasia in patients with MGD (42). It is postulated that altered n-6 polyunsaturated fatty acids such as linoleic acid play a vital role in the etiopathogenesis of MGD (42, 45, 46). Arita et al. concluded that linoleic acid is involved in the pathogenesis of telangiectasia and plugging in MGD and is, therefore, a potential biomarker for MGD diagnosis.

Prostaglandin E2 (PGE2) levels are higher in the tears of patients with MGD than healthy controls (47, 48). To support the fact that PGE2 may differentiate MGD from normal eyes, one study reported that intense pulse light (IPL) treatment changed the concentration of PGE2 in the tears of patients with MGD compared with the control eyes, with the mean concentration of PGE2 being significantly decreased in the treated MGD eyes with a corresponding improvement in clinical signs compared with healthy controls (47). PGE2 may be elevated in other ocular surface diseases aside MGD, and it could be explored as a potential monitoring biomarker for ocular surface inflammation. This could help decipher whether a particular MGD treatment would be effective even before clinical signs and symptoms are ameliorated.

There are around 34 cholesterol esters with carbon numbers ranging from 14 to 34 detected in human meibomian gland epithelial cells. Notwithstanding prevailing conditions, human meibomian gland epithelial cells exhibit a cholesterol ester profile that is around 14.0% saturated, 60.6% monounsaturated, and 25.4% polyunsaturated (49). At the ocular surface in healthy people, the quantity of cholesterol esters is lesser than wax esters (50). Considerably, large differences in cholesterol ester expression are noted in several donors with normal meibum, who have stable tear films (50, 51) implying that cholesterol esters are not likely to confer tear film stability. In MGD, however, it is evident that the cholesterol ester expression decreases, and its molar ratio with wax esters drops further (38, 50). The tear film cholesterol esters have ideal characteristics that permit them to be potent mediators of phase transition and subsequently meibum viscosity (52). Due to the increase in saturation (five times) of cholesterol esters relative to wax esters (53), a decrease in cholesterol esters may inversely affect the meibum’s melting point, thereby increasing its viscosity. This assertion has been confirmed biophysically, biochemically, and clinically in patients with MGD (2, 49).

Cholesterol esters with the number of carbons ≥24 are not present in culture media, serum, epithelial cells, or other tissues except the sebaceous glands making them promising molecules with the potential for distinguishing disease states as diagnostic biomarkers (49). One study has intimated that cholesterol esters with the number of carbons ≥24 are potential lipid biomarkers of human meibomian gland epithelial cells (49). Meibomian glands epithelial cells are less differentiated and regenerated in MGD (3, 49).

It is evident that cholesterol esters as a functional group of molecules are not the preview of any specific tissue; however, cholesterol esters with ultra-long-chain fatty acids are limited to or present only in meibomian and sebaceous glands (49). To further support the argument that cholesterol esters are potential biomarkers, recent studies by Borchman et al. (50) and Shrestha et al. (38) demonstrated that cholesterol esters (exact species not specified) are reduced in adults with meibomian gland dysfunction (54).

Analysis of human meibum from a patient with MGD and age- and gender-matched subjects revealed severely reduced reservoir of normal meibomian lipids such as cholesteryl esters and wax esters in meibum and tears as well as their distorted molecular profiles (55). Furthermore, a three times increase in free cholesterol to cholesteryl esters ratio and over 20 times increase in the triglycerides fraction over the norm were also observed (55). The pattern did not change during a 1-year period when the patient with MGD was examined three times.

Many a meibum-derived (O-acyl)-omega-hydroxy fatty acids (e.g., 118:2/30:1, 18:1/26:1) have been demonstrated to be linked with tear film thinning, intimating that (O-acyl)-omega-hydroxy fatty acids play vital roles in precorneal tear film stability. However, the biophysics of (O-acyl)-omega-hydroxy fatty acids that makes them potent in mitigating tear film evaporation remains elusive in the current literature. Most of these (O-acyl)-omega-hydroxy fatty acids have one or more double bonds in their acyl chain moiety, and hence, it may be plausible that the degree of unsaturation could be a characteristic of (O-acyl)-omega-hydroxy fatty acids aiding them in their functional roles.

MGD manifests with differential expression of specific (O-acyl)-omega-hydroxy fatty acids in the tear film and meibomian secretion. It is suggested that (O-acyl)-omega-hydroxy fatty acids are involved in the changes occurring in the biochemical profile of the lipid layer of tears in individuals with MGD. It has been unequivocally shown that all (O-acyl)-omega-hydroxy fatty acids except 18:2/16:2 in meibum and tears are lesser in MGD compared with healthy controls (56). This implies that (O-acyl)-omega-hydroxy fatty acids may be a potential source of molecules to identify biomarkers. A recent study sought to differentiate between wax esters, cholesterol esters and (O-acyl)-omega-hydroxy fatty acids. The abundance of the human precorneal tear film and meibum-derived wax esters and cholesteryl esters were absolutely independent of MGD disease status and precorneal tear film thinning (evaporation) (57). Apparently, the researchers suggest that altered wax esters and cholesteryl esters do not contribute significantly to alterations in tear film dynamics in MGD (57), as shown in the case of (O-acyl) ω-hydroxy fatty acids (58).

Researchers sought the differences in meibomian fatty acid composition in patients with MGD, aqueous deficient dry eye, and healthy subjects (36, 37). Meibomian fatty acids underwent transmethylation and were analyzed *via* gas chromatography and mass spectrometry. Meibomian fatty acids were not different between dry eye and healthy controls but were different in patients with MGD, in whom there was a substantial elevation in the levels of branched-chain fatty acids and lower levels of saturated fatty acids, especially lower levels of palmitic (C16) and stearic (C18) acids (37). Meibomian fatty acid composition, especially the increased branched chains, could be a biomarker for MGD (37, 59). To further support increased branched chains as a potential biomarker, one study compared treatment with minocycline along with lid hygiene and treatment with lid hygiene only (60). It was revealed that a significant reduction in branched-chain meibomian fatty acids occurred in minocycline-treated patients compared with lid hygiene only patients, which also correlated with an increase in tear breakup time (60).

The lipid structural order can be considered lipid fluidity; however, fluidity may have a supplemental mobility component (61). When lipids are ordered, the hydrocarbon chains are closely packed, which amplifies van der Waals interactions between hydrocarbon chains and subsequent *trans* rotamer conformation of carbons (61). When lipids are disordered, their *trans* rotamers are traded for more *gauche* rotamers (61). Besides, the hydrocarbon chain becomes less close and robust, diminishing the van der Waals interactions between hydrocarbon chains. The peak height ratios and saturation contribute to a higher meibum lipid order that accompanies meibomian gland dysfunction and dry eye (61). Hence, the hydrocarbon order contributes to an unstable tear film and may be a biomarker for MGD (61).

## Proteins and Amino Acids in Tears and Meibum as Candidate Biomarkers

### Amino Acids

Amino acids are the building blocks for many transmitter molecules such as neuropeptides and are likely to be upregulated or downregulated when the ocular surface integrity is insulted or when ocular surface homeostasis is interrupted. Tear compounds, including phenylalanine and isoleucine, are elevated in dry eye-associated MGD (DE-MGD) (62). Simultaneously, glycoproteins (not an amino acid) and leucine are decreased in the tears of patients with DE-MGD compared with healthy controls (62). Furthermore, cysteine, valine, arginine, and tryptophan were significantly higher, while leucine and glycine were markedly lower in the tears of patients with DE-MGD than that of the healthy normal controls (62). The observation of tear upregulation of arginine, valine, cysteine, phenylalanine, isoleucine, and tryptophan in the patients with DE-MGD may be due to the vital roles of these molecules in several signaling pathways of ocular surface disease. One cannot rule out the plausibility that a major process such as neuropeptide hydrolysis by neuropeptidase can occur, releasing these amino acids into tears to convert these molecules into others. It is known that phenylalanine overexpression occurring in the tears of patients with DE-MGD shows the proinflammatory response in DE-MGD, as suggested by several authors (62–64). Also, arginine is speculated to modulate T-cell metabolism and cell survival *via* gene expression (65). Since MGD is associated with clinically apparent inflammation, it may not be surprising that amino acids such as arginine and phenylalanine involved in ocular surface inflammation are significantly higher in the DE-MGD tears (62). These molecules provide important avenues to research and discover important MGD biomarkers.

## Receptor and Storage Proteins

Studies have made it clear that aging human meibomian glands exhibit reduced meibocyte differentiation and cell cycling, which is traditionally linked with the pathogenesis of MGD (66). It has been shown that altered peroxisome proliferator-activated receptor-γ (PPARγ) signaling leads to acinar atrophy and is involved in the pathogenesis of age-related hyposecretory MGD (66). A lack of PPAR-γ could contribute to the potential of age-related atrophic processes of the meibomian glands.

Intracellular lipids, after being made *via* a PPAR-γ-dependent pathway, are kept inside specialized compartments that contain the storage molecule referred to as adipophilin or the associated molecule adipose differentiation-related protein (ADRP) (67). ADRP enhances the uptake of long-chain fatty acids, and the presence of these fatty acids promotes and upregulates its production. It implies that in the case of altered meibum production, ADRP production reduces, making it a potential candidate biomarker for MGD (66).

## Cytokines as Potential Biomarkers of Meibomian Gland Dysfunction

Meibomian gland dysfunction is associated with clinically apparent inflammation, and interleukins may be key players in initiating and sustaining inflammation in MGD. The proinflammatory cytokine IL-1β is an inducer of hyperkeratinization in cultured meibomian gland ducts (68). Matrix metalloproteinase-9 levels are increased in the tear film of patients with MGD (69). The activity of matrix metalloproteinase-9, a zinc and calcium ion-dependent enzyme, activates the precursor IL-1β in the extracellular environment (70), a perpetuator of hyperkeratinization in the meibomian glands. This is consistent with the hypothesis put forth by Jester et al. in an expert review that “inflammation-induced hyperkeratinization of the duct caused by the development of dry eye cannot be ruled out as a consequence of environmentally induced changes in meibomian gland function and lipid quality” (71).

Several reports have highlighted increased tear inflammatory cytokines such as IL-17 and IL-6 in patients with dry eye disease owing to MGD (47, 72, 73). IL-6 and IL-17A are reduced in the tears from patients with MGD after intense pulse light treatment, and the levels of both IL-6 and IL-17A in the tears correlated with meibomian gland yield secretion score: meibomian glands yielding liquid secretion (MGYLS), meibomian glands yielding clear secretion (MGYCS), and meibomian gland yield secretion score (MGYSS) at the pre-treatment baselines (47). However, the correlation analysis between the IL-17A and IL-6 and SPEED/OSDI showed no statistical significance. Furthermore, the change in the concentration of IL-6 in tear film correlated with the change in the values of meibomian glands yielding clear secretion (MGYCS) after IPL treatment. This change suggests that the improvement in meibomian glands yielding clear secretion (MGYCS) is likely to result in a difference in the concentration of IL-6 after IPL treatment (47). It has been noted that the reduced rate of IL-6 level was higher than that of IL-17A (−84 vs. −52% at the end of the study) (74) suggesting that IL-6 may be involved in improving the meibomian gland signs after IPL treatment (47). Furthermore, studies have shown that IL-8 levels are different between healthy controls and patients with MGD (75). In an MGD group, IL-10 levels were significantly lower than that found in the aqueous deficient dry eye among Sjogren syndrome patients (75). Studies have shown an increased IL-6, TNF-α, corneal staining, and meibomian gland loss in patients with MGD (76). In contrast, lipid layer thickness was negatively correlated with IL-2 and IL-4 in patients with MGD (76). Researchers have also shown a significant increase in the expression of IL-6 and TNF-α in the tear fluid of the dry eye patients with MGD when compared with dry eye patients without MGD (76). In one study, subjects were divided into two groups: Group I had no or minimal MGD, and group II had grades 2–4 MGD; mean IL-2, IL-6, and tumor necrosis factor (TNF)-α levels in group II were higher than those of group I (77). This indicates increasing interleukin levels with worsening MGD (77). To further affirm the potential utility of interleukins as a monitoring biomarker in MGD, clinical outcomes and tear cytokine levels in patients with MGD were assessed in a clinical trial. The trial consisted of two groups comprising those treated with oral minocycline and artificial tears (Group 1) versus artificial tears only (Group 2). Patients in group 1 showed statistically significant improvement in all clinical signs and symptoms after 1 month and 2 months of treatment compared with group 2. In addition, there was a statistically significant reduction in IL-6, IL-1β, IL-17α, TNF-α, and IL-12 after 2 months of treatment in group 1 compared with group 2 (78).

## Tear Proteins as Potential Biomarkers for Meibomian Gland Dysfunction

Meibomian gland dysfunction is associated with changes in protein constituents in the meibum and subsequently the tear film. In an investigation, it was revealed that several proteins were downregulated in both dry eye and MGD, including prolactin inducible protein (PIP), zinc-α-2-glycoprotein (AZGP1), galectin 7 (LEG7), cystatin S (CST4), actin cytoplasmic 1 (ACTB), lactotransferrin (LTF), cystatin SN (CST1), and mammaglobin-B (SG2A1) (74). However, the magnitude of the downregulation for each pathology was distinct. For instance, LCN1, PIP, LTF, and SG2A1 were substantially reduced in dry eye, while AZGP1, LEG7, CST4, CST1, and ACTB were diminished in MGD (74). In the dry eye and MGD, these proteins’ expression levels were decreased compared with healthy controls (74). These proteins are secreted by the lacrimal glands. They have also been detected in the meibomian gland secretions (35, 79).

Similarly, galectin 7 and cytoplasmic 1 were reduced in dry eye and MGD compared with healthy controls but significantly worse in patients with MGD (18, 21). Another protein, mammaglobin b (SG2A1), is produced less in evaporative dry eye (80). Glycoprotein is the most abundant protein found in normal tears. It was considerably lower in patients with DE-MGD (62). Furthermore, the changes in the levels of annexin A1, clusterin, and alpha-1-acid glycoprotein 1 were different between MGD and healthy controls (23).

Tear lactoferrin levels are not associated with meibomian gland atrophy but are associated with MGD (81). It is suggested that MGD may adversely temper with the palpebral apparatus, which could incite inflammation in ancillary lacrimal glands and impact lactoferrin secretion (81). The lacrimal gland is the primary source of tear lactoferrin, and it is a known biomarker for aqueous deficiency (81). Posterior lid margin redness and telangiectasias that occur in MGD are also markers of inflammation (7), which may seep inflammatory mediators to affect the accessory lacrimal glands of Krause and Wolfring. This phenomenon has entrenched the idea that inflammation impacts adversely on the function of the accessory lacrimal glands. Furthermore, lactoferrin has been found in meibomian secretions based on proteome analysis (35), implying that abnormal meibum secretion may partially account for the reduction in tear lactoferrin concentration, because not all the detected tear lactoferrin may be due to the lacrimal glands. However, additional work is necessary to understand the relationship between MGD and tear lactoferrin levels.

The most overexpressed proteins in the MGD appear to be annexin-a1, clusterin, alpha-1-acid glycoprotein 1, and lactoperoxidase. Lactoperoxidase had its lowest concentration in patients with dry eye compared with healthy controls and patients with MGD. These molecules are involved in oxidative stress, apoptosis, immune response, and keratinocyte differentiation, which points to the principal processes involved in the health and disease of the meibomian glands (82). A panel of thioredoxin, immunoglobulin heavy constant gamma 1, phospholipase A2, serpin family A member 1, secretory leukocyte peptidase inhibitor, and lactoperoxidase could unequivocally distinguish between dry eye disease and MGD (82). Thioredoxin plays roles in immune response, cell-cell signaling, cell proliferation, and redox mechanisms (83). Immunoglobulin heavy constant gamma 1 and secretory leukocyte peptidase inhibitor are also involved in immunological activities (82, 84), while phospholipase A2 is implicated in the inflammation, immune responses, and lipid catabolism (85). Serpin family A member 1 plays roles in acute-phase response and platelet activation (86), and secretory leukocyte peptidase inhibitor is involved in the defense response (87). Tear proteins are altered in MGD and may be a potential source of biomarkers for MGD.

Lipocalin 1 is a primary protein found in the tear film. It can remove phospholipids and fatty acids from the ocular surface, especially the cornea (88). Studies have shown that its concentration is reduced in tear samples from patients with MGD and dry eye (89).

Studies have shown that lipocalin deficiency is associated with MGD (89). It has been reported that tear lipocalin can bind various lipophilic compounds, including fatty acids, arachidonic acid, fatty alcohols, glycolipids, retinol, phospholipids, and cholesterol, with a particular affinity for phospholipids and insoluble long-chain fatty acids (90). In one study, it was shown that mean tear lipocalin concentration in patients with MGD was substantially reduced compared with concentrations in normal controls (89). This may not be the result of decreased tear secretion since the same volume of tears was collected from all patients, and the total tear protein concentration was not different between patients with MGD and normal controls (89). These findings indicate that decreased tear lipocalin concentration in tears is involved in the pathogenesis of MGD meaning that not only changes in the meibomian glands is responsible for the clinical signs and symptoms in MGD (89).

## Keratins as Candidate Biomarkers for Meibomian Gland Dysfunction

Hyperkeratinization is a key feature of obstructive MGD making keratins a potential source of molecules to research and discover important biomarkers of obstructive MGD. It has also been shown that MGD excreta included a 10% increase in the quantity of detectible ductal cytokeratins (91).

Keratins predominantly come from the shedding of keratinized epithelial cells lining the meibomian gland ducts (25). It has been noted that keratin mixes into the tear film’s lipid layer and can destabilize the lipid layer *in vitro* (25, 92). Keratin 10 and keratin 1 are considered to be essential keratinization markers because they are present in terminally differentiated keratinocytes (93). To confirm whether the ductal epithelia’s hyperkeratinization is responsible for the meibomian glands’ ductal obstruction, some researchers studied the expression patterns of Keratin 10 and keratin 1 in the four donors’ meibomian glands (93). The study finding suggested that the central ductal epithelia’s abnormal differentiation and proliferation may be responsible for the ductal obstruction. This study also showed that ductal epithelial abnormal differentiation and proliferation alone, barring any conspicuous hyperkeratinization, can obstruct the meibomian glands (93). The decline in acinar cell proliferation and renewal was purported to be the underpinning instigator of meibomian gland atrophy with aging (93). This evidence shows that overexpression of hyperproliferative keratins in meibum may be potential biomarkers to detect obstructive MGD accurately (93). Again, other researchers have demonstrated that cytokeratins CK1, CK10, CK13, CK14, and CK19 may be candidate biomarkers within human eyelid tissue to uncover the meibomian gland ducts’ keratinization around the mucocutaneous junction (54, 94).

## Cell Morphology and Immunohistochemistry

Meibomian gland dysfunction can impair ductal epithelial cells and subsequently ocular surface epithelial cell morphology and immunohistochemical properties. This makes cell morphology and immunohistochemistry potential avenues to explore candidate biomarkers for MGD. One study recruited 40 healthy subjects and stained (19 soft contact lens wearers and 21 non-contact lens wearers) their lid margin with lissamine green (95). Impression cytology of the upper lid margin of both eyes was collected, fixed, and stained with periodic acid Schiff (PAS) and hematoxylin for cell morphology analysis and immunocytochemistry (95).

Immunocytochemistry following PAS/hematoxylin staining indicated transition in epithelial cell morphology in the marginal conjunctival epithelium, mucocutaneous junction, and squamous epithelium, close to the meibomian gland ducts (95). The authors concluded that impression cytology combined with histochemistry and immunocytochemistry staining would be vital in assessing the lid margin region’s epithelial cells, including the areas adjacent to the meibomian gland openings. Impression cytology and clinical tests could be useful diagnostic biomarkers for MGD and lid wiper epitheliopathy (95).

Studies have also demonstrated that T helper-17-mediated neutrophil influx plays a role in the obstruction of meibomian glands in the murine model of allergic eye disease (AED) and provided evidence for the association between tear neutrophils and MGD severity (96, 97). Utilizing the experimental model of AED and examining human samples with MGD and blepharitis, it became evident that aggregated neutrophil extracellular traps were an etiopathological factor instigating obstructive MGD in mice and humans (97). It has also been shown that ocular discharge from patients with blepharitis contains aggregated neutrophil extracellular traps (97). Besides, MGD-affected human meibomian glands’ ducts are highly congested with aggregated neutrophil extracellular traps. Furthermore, the tear film of patients with MGD has increased neutrophil chemoattractants (C5a, IL-6, IL-8, and IL-18) (97). Blocking aggregated neutrophil extracellular traps formation with peptidyl arginine deiminase type 4 (PADI4) effectively ameliorates MG damage (97). The detection of the neutrophil extracellular traps (NETs) with immunofluorescence analysis of ocular surface discharge has shown the potential role of neutrophil extracellular traps as a biomarker for meibomian gland obstruction (97).

In one study, investigators explored the reliability and utility of *in vivo* corneal confocal microscopy (IVCM)-based immune-cellular metrics of palpebral conjunctival in subjects with MGD (98). Compared with controls, people with MGD had higher conjunctival epithelial immune cells and intraglandular immune cells (98). Both conjunctival epithelial immune cells and intraglandular immune cells negatively correlated with tear breakup time (98). It was apparent in the study that conjunctival epithelial immune cells and intraglandular immune cells increased in highly symptomatic patients with MGD that have minimal corneal staining (98). Conjunctival epithelial immune cells and intraglandular immune cells may provide reliable and clinically relevant metrics of inflammation in symptomatic MGD (98).

## Tear Enzymes as Candidate Biomarkers for Meibomian Gland Dysfunction

Enzymes are important in meibum synthesis; however, in MGD a couple of enzymes may be upregulated or downregulated due to the changes in meibum or stasis of gland content. A recent study on tears proteomic analysis showed different sets of proteins that differentiate between MGD and dry eye comprising antileukoproteinase, phospholipase A2, and lactoperoxidase (23). Another study recruited three groups comprising MGD, aqueous deficient dry eye groups, and normal healthy controls. Transforming growth factor-2 and matrix metalloproteinase-9 were expressed significantly higher in both patient groups than in controls. However, Sjogren-related dry eye patients showed a higher expression than the MGD group (99). Again, Solomon et al. discovered that tear film matrix metalloproteinase-9’s activity levels were significantly higher in patients with MGD than in healthy controls (69). Furthermore Moon et al. conducted a retrospective case series study with a total of 48 eyes of 24 patients with a diagnosis of moderate to severe MGD undergoing a single session of lid debris debridement using the BlephEx combined with meibomian gland expression (100). There were significant improvement in the ocular surface staining scores, lid margin findings (lid thickness and telangiectasia), tear breakup time, meibomian gland function, and symptoms (100). This was accompanied by substantial matrix metalloproteinase-9 immunoassay positivity rate reduction from 4 weeks after treatment (100). This supports the potential use of matrix metalloproteinase-9 as a monitoring biomarker in MGD.

The basis for the increased programmed cell death of Cu, Zu-superoxide dismutase-1 knockout meibomian glands’ cells is due to the increased apoptosis instigated by cytokines such as IL-6, in addition to the mitochondrial ultrastructural changes (101). Investigation into the alterations of meibomian glands in Cu, Zu-superoxide dismutase-1 knockout mice showed a buildup of large lipid droplets and oxidative stress marker staining in the acinar epithelium, indicating the vital roles of reactive oxygen species in the pathogenesis of MGD and the fact that Cu, Zu-superoxide dismutase-1 absence could signal the presence of MGD (101, 102). Tear and serum IL-6 and TNF-α levels increased in the 10- to 50-week-old Cu, Zu-superoxide dismutase-1 knockout mice (101). This implies that reactive oxygen species may have a potential role in the pathogenesis of MGD.

Despite the vital role of the meibomian secretion, its biosynthesis and the roles of specific lipid constituents remain elusive in the current literature. There is considerable evidence that the genetic deletion of Acyl-CoA: wax alcohol acyltransferase 2 (AWAT2) instigates meibomian gland obstruction (103). The constituents of meibomian lipids isolated from Acyl-CoA: wax alcohol acyltransferase 2 negative mice showed the absence of wax esters, but rather an upsurge and overproduction of cholesteryl esters (103). Although the number of fatty acids remained unchanged, an approximately 8 times higher quantity of cholesteryl esters occurs in the meibum (103). The increase in cholesterol esters alters meibum viscosity, melting point, and phase transition. In another study, researchers created single and double knockout mice for the two acyl-CoA wax alcohol acyltransferases (Awat1 and Awat2) and studied their ocular surface changes and meibum components (104). Awat2 knockout mice and Awat1 and Awat2 double knockout mice expressed severe dry eye with MGD, whereas Awat1 knockout mice had only mild dry eye (104). This implies that measuring Awat2 levels could be a good indicator of MGD. It has become increasingly known that the ablation of several key genes of meibogenesis pertaining to omega oxidation, fatty acid elongation, and esterification into wax esters resulted in predictable changes in the meibum lipid profiles and caused severe abnormalities in meibomian gland morphology and physiology.

There is considerable evidence of cholesterol ester-depleted meibum losing its integrity in the formation of thin and continuous lipid devoid of high fragmentation in mice (105). These observed manifestations can be interpreted as evidence of its lost ability to form the tear film lipid layer and as a result not able to mitigate corneal nociceptors’ firing (105). However, the mechanism of these observed changes in mice needs further clarification requiring additional work in future experiments. This change in meibum caused by sterol O-acyltransferase 1-ablated mice mirrored many exact characteristics of human MGD. Ablating sterol O-acyltransferase 1 resulted in almost all loss of cholesterol esters in meibum and gradual mass gathering of their precursor—free cholesterol—in large quantities, virtually replacing very, extremely, and ultra-long cholesterol esters and becoming the main components of abnormal meibum (105).

Apart from the “*ductal centric*” hypothesis that comprises epithelial hyperkeratinization causing obstructed meibomian gland orifice and promoting stasis of meibomian gland contents, there is also “meibocyte centric” hypothesis that involves mechanisms or processes that modulate differentiation and renewal of meibocytes that affect meibum quality, meibum synthesis, and acinar atrophy without any ductal epithelium changes (3). Simply put, loss of meibocyte differentiation impacts the ability of meibocytes to synthesize meibum leading to a hyposecretory MGD. Meibocytes transit through the acinus, from the basal compartment to the disintegrating compartment, which is crucial for normal meibomian gland function (106), because continuous basal meibocyte renewal is needed for normal meibomian gland function. During this process, the meibocytes go through maturation stages (basal, differentiating, mature, and hypermature) that can be differentiated morphologically (2). Hence, markers of meibocyte differentiation could serve as a biomarker for MGD.

There is evidence that leucine-rich repeats and immunoglobulin-like domains protein 1 (Lrig1) and *deoxyribonuclease-2* (DNase2) serve as biomarkers for human meibomian gland progenitor and differentiated cells, respectively. Lrig1 is present in the meibomian gland basal epithelial cells in the acinar periphery (107). DNase2 is also present in the differentiated epithelial cells of the meibomian gland central acinus (107). It is postulated that Lrig1 and DNase2 could be biomarkers for progenitor and differentiated cell populations in the human meibomian glands. The reason for this line of argument is that Lrig1 is a biomarker for proliferating progenitor cells in the pilosebaceous unit and that these precursor cells become differentiated into meibomian epithelial cells (meibocytes) (107). Furthermore, lysosomal DNase2 is noted for activating the nuclear degeneration and holocrine secretion of sebocytes (107). It has been shown that DNase2 is produced only in the lipid-containing differentiated cells of the human meibomian glands (107). This evidence suggests that Lrig1 and DNase2 may serve as candidate biomarkers for MGD (107).

## Cadherins as Candidate Biomarkers for Meibomian Gland Dysfunction

Cadherins (subtypes of classical cadherins, E-, N-, and P-cadherin) are cell surface glycoproteins involved in calcium-dependent heterotypic and homotypic cell-cell adhesion (54). In one study, it was demonstrated that N-cadherin occurs in all layers of the cells in the skin epidermis, mucocutaneous junction, and conjunctiva (108). It is also present in the acini and ductal epithelium of the meibomian glands (54, 108). Concerning MGD, the epithelium around the eyelid margin and the mucocutaneous junction are tissues of particular interest because confirmed knowledge of their interactions is fundamental in this condition’s noticeable pathological changes (54). These pathological changes, including keratinization of the meibomian glands, are believed to be the main pathomechanism for MGD making cadherins a potential group of molecules to search for candidate biomarkers. When cadherin was studied to investigate the control of cell adhesion in human meibomian gland epithelial cells using the new human *ex vivo* slice culture model, it was demonstrated that cell adhesion is maintained differently in meibomian gland cells and that E-cadherin is essential for meibomian gland function (108).

Lipid synthesis and secretion were unaffected in meibomian glands from desmoglein-3-deficient mice; hence, an *ex vivo* slice culture model of human eyelids was established to permit studies in a favorable physiological environment (108). E-Cadherin is essential for meibomian gland function, which is revealed in studies using the new human *ex vivo* slice culture model (108). Most meibocytes expressed desmosomal cadherins, including desmocollins, desmoglein (Dsg), and E-cadherin (Ecad), as the primary adhesion molecule of the adherens junction (AJ) (108, 109).

## Carbohydrates and Other Molecules as a Potential Source of Biomarkers for Meibomian Gland Dysfunction

It has been demonstrated that glucose concentration is significantly higher in the DE-MGD tears than in healthy controls (62). This is consistent with the reported increase in the frequency of MGD in patients with diabetes compared with patients without diabetes (110). Simultaneously, acetate, a metabolite of glucose metabolism, is significantly reduced in the tear film from the patients with DE-MGD than healthy controls (62, 66). Acetate is a molecule that supports acetyl-coenzyme A metabolism and thus lipogenesis and protein acetylation (111). The reduced levels of acetate in tears of DE-MGD implies that any alteration in ocular surface homeostasis that occurs in MGD might upregulate glucose metabolism in the ocular surface cells. This situation presents as an increased glucose level and reduced acetate concentration, which is commensurate with increased glucose metabolism. These molecules and other end-products of excess glucose metabolism such as advanced glycation end-products in tears can be explored as potential risk biomarkers for MGD, especially in patients with diabetes.

## Conclusion

Meibomian gland dysfunction studies have revealed several molecules, including but not limited to proteins, amino acids, carbohydrates, and enzymes that may be potential candidate biomarkers for this prevalent ophthalmic condition, which also doubles as the leading cause of dry eye disease. This is because these molecules are either elevated or reduced in patients with MGD than in healthy controls. Some show apparent differences in tear and meibum concentration between dry eye disease and MGD. It is of utmost importance to recognize that MGD is a complex condition, making it difficult to distinguish patients using single biomarkers. Therefore, multiple biomarkers forming a multiplex panel may be required.

## Author Contributions

The author confirms being the sole contributor of this work and has approved it for publication.

## Conflict of Interest

The author declares that the research was conducted in the absence of any commercial or financial relationships that could be construed as a potential conflict of interest.

## Publisher’s Note

All claims expressed in this article are solely those of the authors and do not necessarily represent those of their affiliated organizations, or those of the publisher, the editors and the reviewers. Any product that may be evaluated in this article, or claim that may be made by its manufacturer, is not guaranteed or endorsed by the publisher.
